# Prevalence of white matter pathways coming into a single white matter voxel orientation: The bottleneck issue in tractography

**DOI:** 10.1002/hbm.25697

**Published:** 2021-12-17

**Authors:** Kurt G. Schilling, Chantal M. W. Tax, Francois Rheault, Bennett A. Landman, Adam W. Anderson, Maxime Descoteaux, Laurent Petit

**Affiliations:** ^1^ Department of Radiology & Radiological Science Vanderbilt University Medical Center Nashville Tennessee USA; ^2^ Vanderbilt University Institute of Imaging Science Vanderbilt University Medical Center Nashville Tennessee USA; ^3^ Cardiff University Brain Research Imaging Centre (CUBRIC), Cardiff University, Cardiff, United Kingdom Cardiff UK; ^4^ Image Sciences Institute University Medical Center Utrecht Utrecht The Netherlands; ^5^ Department of Electrical Engineering and Computer Science Vanderbilt University Nashville Tennessee USA; ^6^ Department of Biomedical Engineering Vanderbilt University Nashville Tennessee USA; ^7^ Sherbrooke Connectivity Imaging Lab (SCIL), Computer Science Department Université de Sherbrooke Sherbrooke Quebec Canada; ^8^ Groupe d'Imagerie Neurofonctionnelle Institut Des Maladies Neurodégénératives, CNRS, CEA University of Bordeaux Bordeaux France

**Keywords:** bottleneck, crossing fibers, fiber pathways, tractography, tractometry, white matter

## Abstract

Characterizing and understanding the limitations of diffusion MRI fiber tractography is a prerequisite for methodological advances and innovations which will allow these techniques to accurately map the connections of the human brain. The so‐called “crossing fiber problem” has received tremendous attention and has continuously triggered the community to develop novel approaches for disentangling distinctly oriented fiber populations. Perhaps an even greater challenge occurs when multiple white matter bundles converge within a single voxel, or throughout a single brain region, and share the same parallel orientation, before diverging and continuing towards their final cortical or sub‐cortical terminations. These so‐called “bottleneck” regions contribute to the ill‐posed nature of the tractography process, and lead to both false positive and false negative estimated connections. Yet, as opposed to the extent of crossing fibers, a thorough characterization of bottleneck regions has not been performed. The aim of this study is to quantify the prevalence of bottleneck regions. To do this, we use diffusion tractography to segment known white matter bundles of the brain, and assign each bundle to voxels they pass through and to specific orientations within those voxels (i.e. fixels). We demonstrate that bottlenecks occur in greater than 50‐70% of fixels in the white matter of the human brain. We find that all projection, association, and commissural fibers contribute to, and are affected by, this phenomenon, and show that even regions traditionally considered “single fiber voxels” often contain multiple fiber populations. Together, this study shows that a majority of white matter presents bottlenecks for tractography which may lead to incorrect or erroneous estimates of brain connectivity or quantitative tractography (i.e., tractometry), and underscores the need for a paradigm shift in the process of tractography and bundle segmentation for studying the fiber pathways of the human brain.

## Graphical abstract

"Bottleneck" pose a challenge for diffusion tractography, and hinder our ability to accurately map the structural connections of the brain. We demonstrate that bottlenecks occur in greater than 50–70% of fixels in the human brain white matter, and find that all projection, association, and commissural fibers contribute to, and are affected by, this phenomenon. These results emphasize the use of caution when interpreting quantitative diffusion magnetic resonance imaging connectomics results, and underscore the need for a paradigm shift in the process of tractography for studying the fiber pathways of the human brain.

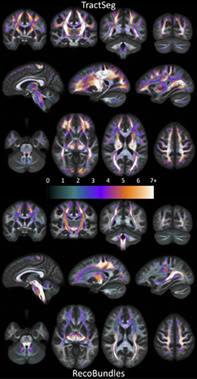

## 1 INTRODUCTION

Two paths diverged from a single orientation,

And streamlines could not travel both

Else it be a false positive, long it stood

And looked down one as far it could

To which cortex should it approach?

Diffusion magnetic resonance imaging (MRI) fiber tractography is currently the only tool to map the long‐range structural brain connectivity in vivo. However, there are a number of limitations and ambiguities that affect the ability of tractography to accurately map the connections of the brain (K. G. Schilling, Daducci, et al., 2019). At the voxel level, significant attention has been given to the “crossing fiber problem” (Alexander & Seunarine, 2010; Tournier, 2010; Tuch, Reese, Wiegell, & Wedeen, 2003). This problem typically refers to the situation when two or more *differently oriented* fiber bundles are located in the same imaging voxel, which causes a partial volume effect that can lead to ambiguous or incorrect estimates of fiber orientation and subsequent failure of tractography (Wheeler‐Kingshott & Cercignani, 2009). Crossing fibers have been shown to occur in a majority of the voxels in the brain (Behrens, Berg, Jbabdi, Rushworth, & Woolrich, 2007; Jeurissen, Leemans, Tournier, Jones, & Sijbers, 2013), and for the last decade the crossing fiber problem has been cited as the major limitation that diffusion tractography faces, with a vast number of algorithms (Daducci et al., 2014) and papers referring to this problem (Alexander & Seunarine, 2010; Behrens et al., 2003; Behrens et al., 2007; Descoteaux, Deriche, Knosche, & Anwander, 2009; Donahue et al., 2016; Dyrby et al., 2007; Dyrby, Innocenti, Bech, & Lundell, 2018; Jeurissen, Descoteaux, Mori, & Leemans, 2019; Knösche, Anwander, Liptrot, & Dyrby, 2015; Mani, Jacob, Guidon, Magnotta, & Zhong, 2015; Poulin, Jörgens, Jodoin, & Descoteaux, 2019b; Rheault, Poulin, Valcourt Caron, St‐Onge, & Descoteaux, 2020; Schilling et al., 2017; K. G. Schilling, Daducci, et al., 2019; K. G. Schilling, Janve, et al., 2018; Schilling, Nath, et al., 2019; Tax et al., 2015; Tournier, 2010; Tournier, Calamante, Gadian, & Connelly, 2004; Tuch et al., 2003; H. Zhang, Dyrby, & Alexander, 2011). The identification and characterization of this problem led to a fundamental paradigm shift in diffusion processing, moving the field beyond classical diffusion tensor imaging, and has led to the development of a number of algorithms capable of resolving crossing fibers (Alexander & Seunarine, 2010; Ning et al., 2015; Tournier, 2010; Tournier et al., 2004; Tournier, Calamante, & Connelly, 2007; Tuch et al., 2003).

A more recently described limitation of fiber tractography is the “bottleneck problem” (Maier‐Hein et al., 2017). In contrast to the crossing fiber problem, bottlenecks occur at a *global level* when multiple fiber populations converge toward a narrow region, temporarily aligning and sharing the *same orientation* and trajectory, before re‐emerging from the bottleneck region (Rheault et al., 2020; K. G. Schilling, Daducci, et al., 2019). Current tractography algorithms cannot adequately choose the correct pathway upon re‐emerging, which leads to generation of a potentially large number of false positive pathways (Maier‐Hein et al., 2017), and limits the ability to use tractography as a tool to explore potential connections and fiber pathways of the brain. While significant efforts have gone into solving the crossing fiber problem, the bottleneck problem has received far less attention. A thorough characterization and investigation of bottleneck locations and prevalence may highlight the extent of this problem, and much like the crossing fiber problem, cause a paradigm shift in tractography in order to solve this issue.

In this work, we utilize well‐known and well‐characterized white matter fiber bundles extracted using automated tools, to quantify how often they overlap within the same imaging voxels, and also how often the overlap occurs within the same voxel while also sharing the same dominant orientation. These locations represent known bottleneck regions for tractography, and indicate areas in the brain where a number of white matter pathways converge, and where tractography may lead to incorrect or erroneous estimates of brain connectivity.

### 1.1 Nomenclature

Here, we aim to clarify nomenclature that will be used in this study to describe our methodology and results. First, a *bundle*, or fiber bundle, is a group of streamlines that is created from a diffusion MRI dataset and is intended to represent a specific white matter pathway of the brain (i.e., a group of axons that connect specific brain regions, also called fiber tracts or fasciculi). Bundles, then, contain streamlines with start and end points generally belonging to the same brain territories, respectively. In this study, we create bundles using two common white matter atlases and tractography dissection techniques that are informed by prior anatomical knowledge and contain pathways for which there is extensive evidence of their existence. Thus, in this study, we are analyzing only *anatomically plausible* bundles, and quantification of the prevalence of bottleneck regions in this study is likely a lower bound of the occurrence of this problem.

Next, a *voxel* represents information in three‐dimensional space. In MRI, the size of voxels is on the order of millimeters, whereas axons of the brain have diameters on the scale of micrometers, and a single voxel can contain hundreds of thousands of axons. In this work, we describe the directionality of axons within a voxel with the *fiber orientation distribution* (FOD). The FOD is a continuous function over a sphere and can be visualized as a histogram on the 2‐sphere, where peaks, or local maxima, are assumed to point parallel to the direction of axons. The FOD can be segmented into discrete elements based on peaks, or lobes, that are considered to be representative of a specific orientation of a set of axons within each voxel. These fiber elements are referred to as *fixels* (Dhollander et al., 2021; D. A. Raffelt et al., 2017), and are parameterized by the mean orientation of fibers within the lobe of the FOD. Thus, there can be multiple fixels in a single voxel, with the advantage that we can now assign a specific property or index to each fixel, or orientation, within a voxel. It is important to note that segmentation of a continuous FOD is just one way in which fixels can be obtained, and there are several descriptions of axon directionality that can be used to characterize fixels within voxels (Dhollander et al., 2021).

**FIGURE 1:**
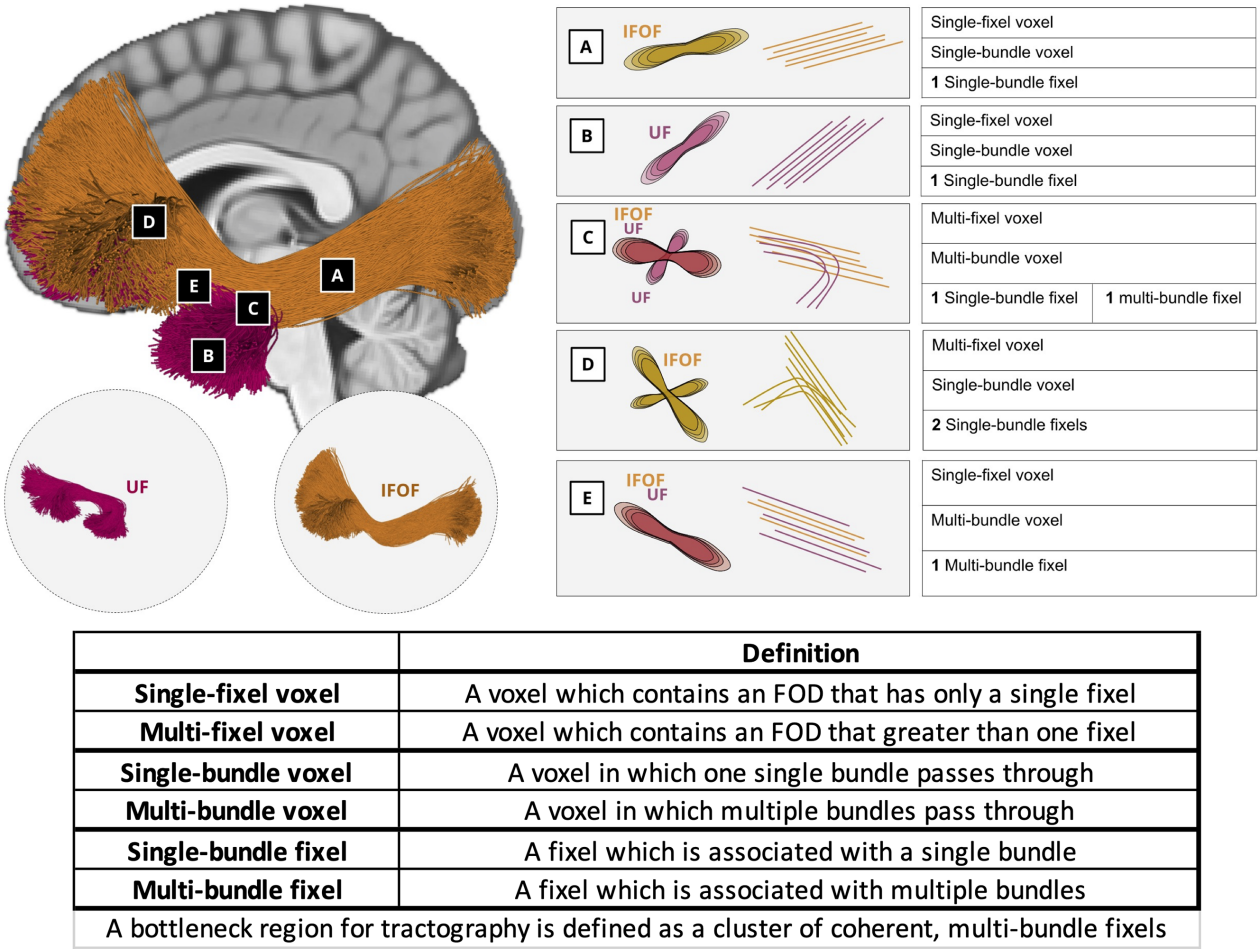
Nomenclature. Two bundles, the UF and IFOF, are used to highlight classifications of voxels (a–e), and fixels within the voxels. Voxels in a and b are examples of single‐fixel voxel and single‐bundle voxels and also single‐bundle fixel. Because the UF and IFOF diverge in Voxel c, this is an example of a multi‐fixel voxel and multi‐bundle voxel, with one fixel classified as a single‐bundle fixel and the other a multi‐bundle fixel. Voxel d highlights the fanning of the IFOF, which results in a multi‐fixel voxel and single‐bundle voxel, and both fixels are single‐bundle fixels. Finally, both the IFOF and UF pass through voxel E following the same orientation, thus Voxel e is a single‐orientation voxel but multi‐bundle voxel, and also a multi‐bundle fixel. This fixel, and thus also this voxel, represents a bottleneck for tractography

For this study, it is necessary to clarify or define six classifications, written out and displayed as a cartoon in Figure 1. If a voxel has only a single fixel (here, a single peak in the FOD) we classify it as a *single‐fixel voxel*, and if it has greater than one fixel we call this a *multi‐fixel voxel* (Jeurissen et al., 2013). In contrast to simply counting fixels, we also count the number of bundles passing through the same voxel, then characterizing the voxel as either a *single‐bundle voxel* or a *multi‐bundle voxel*. Similarly, we count the number of bundles associated with each fixel and characterize the fixel as a *single‐bundle fixel* or a *multi‐bundle fixel*. As described above, a fixel is usually used to describe a single fiber bundle element. However, we hypothesize that a single fixel may be associated with several bundles, thus creating bottlenecks for tractography.

### 1.2 Bottlenecks and when they become a problem

**FIGURE 2:**
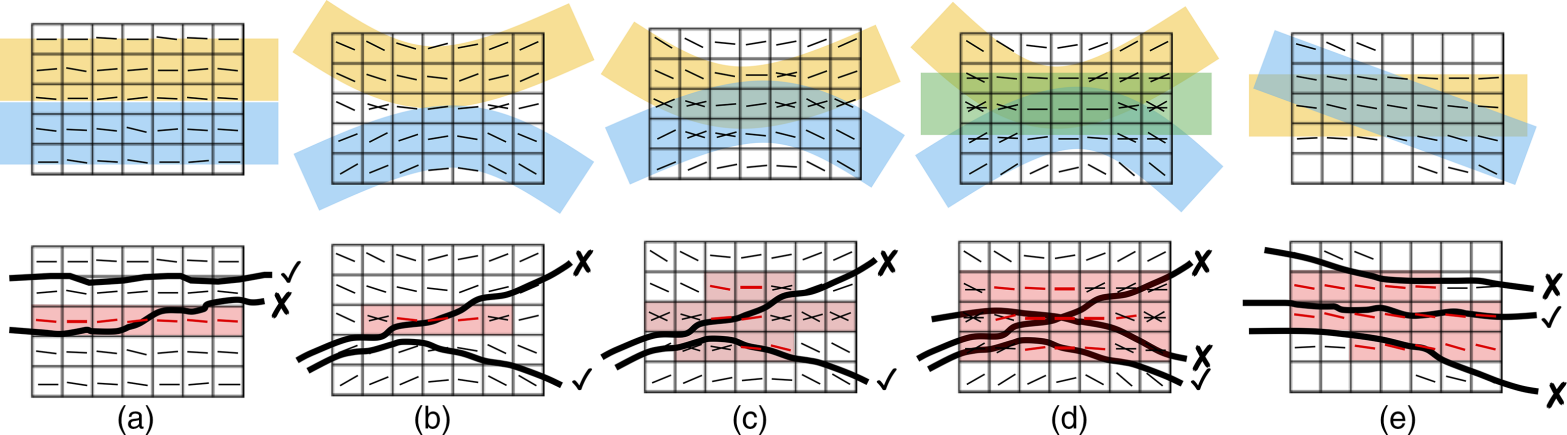
Bottlenecks and when they become a problem. Anytime two connections of the brain start and end at different locations, but at some point, are within the same voxel (spatial partial‐volume), and share an orientation not resolvable by diffusion (angular partial‐volume), diffusion tractography faces a bottleneck. Cartoon examples illustrate increasing levels of pathway complexity: parallel pathways (a), kissing or touching pathways (b), two overlapping pathways (c), three overlapping pathways (d), or bundles crossing at sharp angles not resolvable by diffusion techniques (e). Multi‐bundle voxels are shaded red, and multi‐bundle fixels are also colored red. Bottleneck regions are apparent as spatial clusters of multi‐bundle fixels. In these regions, bundles run nearly parallel, and as tractography streamlines enter and exit these regions they may generate either valid (✓) or invalid (X) connections

Based on this nomenclature, we then define *bottleneck regions* as spatial clusters of coherent multi‐bundle fixels (Figure 2). To understand this definition, it is important to establish *when* bottlenecks become a problem for diffusion MRI. Much like the crossing fiber problem, the bottleneck problem is strongly related to the concept of partial volume effects. As described above, for crossing fibers, or here multi‐fixel voxels, the partial volume effect occurs when two or more differently oriented fiber bundles are located within the same imaging voxel, and the ability to resolve this is limited by the *angular* resolution of the imaging and processing methods. Bottlenecks then occur due to limited *spatial* and *angular* resolution. If we consider the extreme case of deriving an orientation estimate for every point in three dimensional (3D) space (approaching the size of individual neurons), we could unambiguously generate streamlines that follow individual neurons of fiber pathways to form valid connections. However, imposing a voxel grid upon this system causes partial volume effects that are dependent upon the voxel size, the size, and configuration of fiber pathways, and fiber pathways spatial relationship to each other.

In a simple case, two pathways may run parallel and adjacent to each other, with no inter‐digitation of fibers (Figure 2a). In this case, a voxel grid imposed on this system would result in a number of voxels and fixels, which contain fibers from both bundles due to partial volume effects at their interface only. Streamlines within this line/plane of voxels may be able to jump from one pathway to another, despite clear separation in space and unambiguous orientation estimates, although this effect will only occur at the interface. Next, two pathways with no inter‐digitation of fibers may each bend slightly, and briefly touch, or “kiss,” at one location (Figure 2b). Here, partial volume effects will occur within the voxel at their interface. Depending on the curvature of these pathways, the angular resolution may resolve multiple fiber orientations, but even so, false positive pathways may be created as streamlines jump from one pathway to the next. Third, these curved bundles may overlap or inter‐digitate in space (Figure 2c). Regardless of whether the angular resolution is able to separate orientation, any streamline entering this overlap is susceptible to false positive pathways. Finally, this situation becomes more complicated with larger spatial overlap, and the overlap of more than two pathways (Figure 2d), or bundles crossing at angles not distinguishable with diffusion MRI acquisition and reconstruction conditions (Figure 2e), creating a large combinatorial number of trajectories that streamlines may follow. Thus, all of these situations create bottlenecks due to partial volume effects, and will result in spatially coherent clusters of multiple bundles within voxels, and worse, multiple bundles associated with a single fixel. In summary, anytime two connections of the brain start and end at different locations, but at some point, are within the same voxel (spatial), and share an orientation not resolvable by diffusion (angular), diffusion tractography faces a bottleneck.

Diffusion models capable of resolving crossing fibers were pursued in order to be able to disentangle different bundles traversing a single voxel; however, it is clear that if there are multiple bundles traversing a single voxel that cannot be distinguished based on their local orientation alone, then no amount of sophistication in diffusion models can resolve the ill‐posedness of tractography. Thus, bottleneck regions are a problem that affects all applications of tractography. In studies of the human brain connections (i.e., connectomics) bottlenecks will lead to false positive connections and biased subsequent connectome quantifications. In studies of specific fiber pathways partial volume effects with other bundles will hinder the attribution of microstructural or geometrical features to these pathways, as well as identification of spatial location and connectivity profiles. Finally, identification of pathways for neurosurgical applications will also be susceptible to partial volume effects with other bundles that share the same space and orientation with the pathway of interest.

## 2 METHODS

### 2.1 Data

We utilized data from 25 healthy subjects in the Human Connectome Project (HCP) S1200 release (Van Essen et al., 2013). The HCP protocol (custom 3T Siemens Skyra) included T1‐weighted images acquired using a 3D MPRAGE sequence (TE = 2.1 ms, TR = 2,400 ms, flip angle = 8°, FOV = 224 × 224 mm, acquisition, voxel size = 0.7 mm isotropic). Diffusion images were acquired using a single‐shot EPI sequence, and consisted of three *b*‐values (*b* = 1,000, 2,000, and 3,000 s/mm^2^), with 90 directions per shell, and 18 *b* = 0 volumes (TE = 89.5 ms, TR = 5,520 ms, slice thickness = 1.25 mm, flip angle = 78°, FOV = 210 * 180, voxel size = 1.25 mm isotropic). Data preprocessing included correction for susceptibility distortions, subject motion, and eddy current correction. We have chosen HCP data because of its high spatial resolution and multi‐shell acquisition to both minimize spatial partial volume effects and minimize angular resolution partial volume (better resolve multi‐fixel voxels).

### 2.2 Processing

Quantification of fixels per voxel, bundles per voxel, and bundles per fixel was performed in individual subject‐specific space for all white matter. Additionally, a subject‐specific template was created for qualitative visualization, and identification of bottleneck regions across a population.

All processing was performed with the MRtrix3 software package (Tournier et al., 2019). Multi‐shell, multi‐tissue constrained spherical deconvolution (*dwi2fod*) (Jeurissen, Tournier, Dhollander, Connelly, & Sijbers, 2014) was used to estimate the white matter FOD for each subject using a group averaged white matter response function computed using the “*dhollander*” algorithm (i.e., a multi‐tissue response function computed using unsupervised 3‐tissue response function estimation; Dhollander, Raffelt, & Connelly, 2016). Next, we generated the study‐specific unbiased FOD template (*population_template*; D. Raffelt et al., 2011) for population‐based analysis and visualization.

From the subject‐specific FODs, we extracted peaks (*sh2peaks*) (Jeurissen et al., 2013) and fixels (*fod2fixel*), (D. Raffelt et al., 2012; R. E. Smith, Tournier, Calamante, & Connelly, 2013) with an absolute peak threshold of 0.1 in order to remove spurious peaks. We then counted the number of fixels (i.e., lobes of the FOD) and defined those voxels, which have only a single peak to be a *single‐fixel voxel*, and those with greater than one peak to be *multi‐fixel voxels*. For visualization and assessment of spatial consistency across a population, the scalar maps of fixels‐per‐voxel were spatially transformed to template space and averaged across the population space. Additionally, the population FODs were segmented for fixel‐based visualizations in template‐space (see Section 2.4). Note, again, that we have chosen to use the FOD (and subsequent fixel segmentation) to characterize directionality of axons within a voxel, however, a number of reconstruction approaches (Aganj et al., 2010; Behrens et al., 2003; Sotiropoulos, Behrens, & Jbabdi, 2012; Tournier et al., 2004) may have been used to derive fixel‐based descriptions within each voxel.

### 2.3 Bundle segmentation

We utilized two common, automated, pipelines for white matter bundle extraction, Recobundles (Garyfallidis et al., 2018) and TractSeg (Wasserthal, Neher, & Maier‐Hein, 2018a; Wasserthal, Neher, Hirjak, & Maier‐Hein, 2019). These are both informed by prior anatomical knowledge in order to generate bundles representative of well‐characterized, and well‐validated, white matter pathways of the brain (pathways and acronyms given in Appendix). All analysis is performed separately for both techniques to show that results generalize across slight deviations in the number and definitions of white matter pathways, and the techniques used to extract them.

Recobundles is based on an atlas of 78 bundles (Yeh et al., 2018), although 12 bundles are cranial nerves outside of the cerebrum and brainstem. Whole brain tractography was performed using anatomically constrained probabilistic tractography with the iFOD2 propagation algorithm (tckgen), to generate 25 million streamlines, which were filtered based on the diffusion signal using the SIFT algorithm (R. E. Smith et al., 2013) (tcksift) to 2 million streamlines. Bundle recognition was performed following streamline linear registration to the HCP842 (Yeh et al., 2018) bundle template (dipy_slr) and bundle recognition using default parameters of the RecoBundles algorithm (Garyfallidis et al., 2018).

TractSeg (Wasserthal et al., 2018a) is a tool based on convolutional neural networks that is trained to create tract orientation maps and segmentations of end regions, which can be used to perform probabilistic bundle‐specific tractography (Wasserthal et al., 2019). We implemented the processing pipeline provided at (https://github.com/MIC-DKFZ/TractSeg) with the MRtrix‐derived FODs as input, in order to generate 72 bundles per subject. The primary outputs of TractSeg were binary bundle segmentations and tract‐orientation‐maps, which could be used directly to delineate voxels and fixels associated with each bundle. However, we found that performing tractography on the tract‐orientation maps to create a bundle‐specific tractogram (and following the processing described in Section 2.4) resulted in more specific delineations of the bundles. We chose to utilize the bundle‐specific tractograms directly in order to ensure a conservative estimate (i.e., underestimation) of the prevalence of the bottleneck problem.

### 2.4 Assigning bundles to voxels and fixels

**FIGURE 3:**
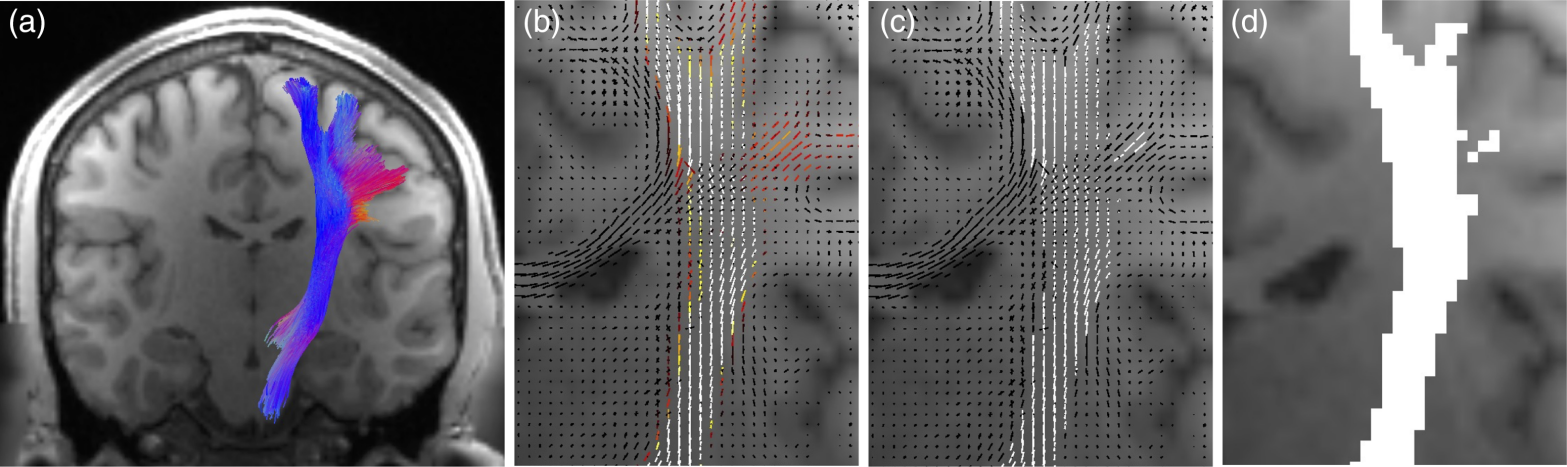
Assigning bundles to voxels and fixels. Each segmented white matter bundle (a) was assigned to each fixel by counting the number of streamlines aligned with each fixel to create fixel‐density map (b) which was thresholded to generate the binary fixel‐based profile of each bundle (c). This allows us to query the number of known bundles per fixel. Next, this map was projected to the voxel level, and binary voxel‐based profiles of each bundle (d) were generated, which allows us to query the number of known bundles per voxel. White matter bundles were derived from TractSeg (*N* = 72 bundles) and Recobundles (*N* = 66 bundles)

The generation of streamlines and subsequent bundle segmentation was performed in individual subject space. Figure 3 visualizes the procedure used to assign bundles to voxels and bundles to fixels. For each bundle (Figure 3a; *N* = 66 RecoBundles; *N* = 72 TractSeg), a fixel density map (Figure 2b) was created (tck2fixel) by counting the number of streamlines that are most closely aligned to each fixel. Fixel‐density maps were thresholded at 5% of the maximum density in order to create binary segmentations indicating the fixel‐wise profile of each bundle (Figure 2c), which associates each bundle with a fixel. Now, the number of bundles per fixel can be counted. Once the number of bundles per fixel is quantified, we then projected this information to the voxel level, as the sum of the number of bundles of all fixels within a voxel (ensuring that each bundle is counted only once with the possibility that it may be assigned to multiple fixels within a voxel) in order to quantify the number of known bundles per voxel (this projection operation is visualized in Figure 2d, for a single pathway). Now, for all subjects, the number of known bundles per voxel, as well as the number of known bundles per fixel, is quantified in template space. Results are quantified for all subjects, and limited to only white matter of the brain as segmented using the “5ttgen fsl” algorithm (S. M. Smith, 2002; S. M. Smith et al., 2004; R. E. Smith, Tournier, Calamante, & Connelly, 2012). Additionally, for visualization and assessment of spatial consistency across a population, streamlines were spatially transformed to template space, assigned to bundles and fixels in template space, and averaged across the population.

## 3 RESULTS

### 3.1 Bottleneck prevalence

**FIGURE 4:**
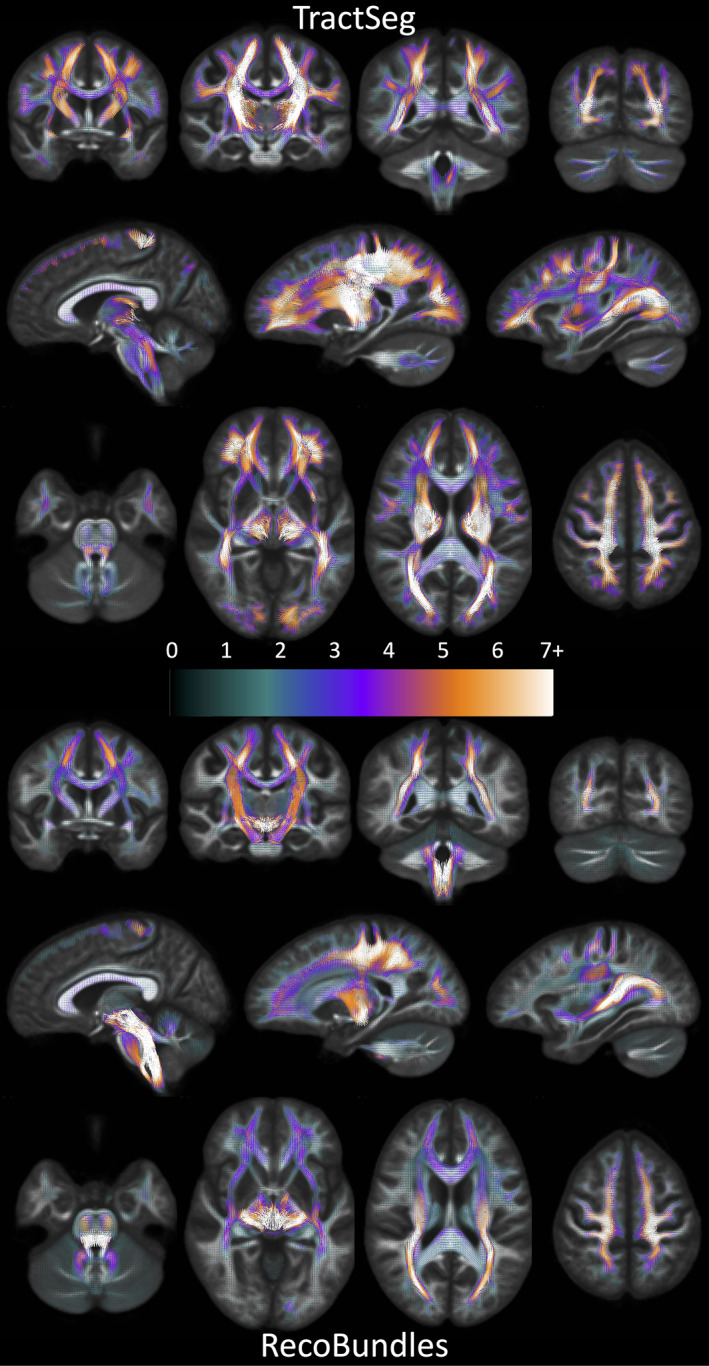
There is a high prevalence of bundles assigned to each fixel in the brain. Fixels, in template‐space, are shown as vectors, colored by the number of associated bundles, and averaged across the population (note continuous color‐map due to population‐averaging). TractSeg results are shown on top, Recobundles on bottom. Fixels with more than one bundle traversing through them represent bottleneck regions for tractography

**FIGURE 5:**
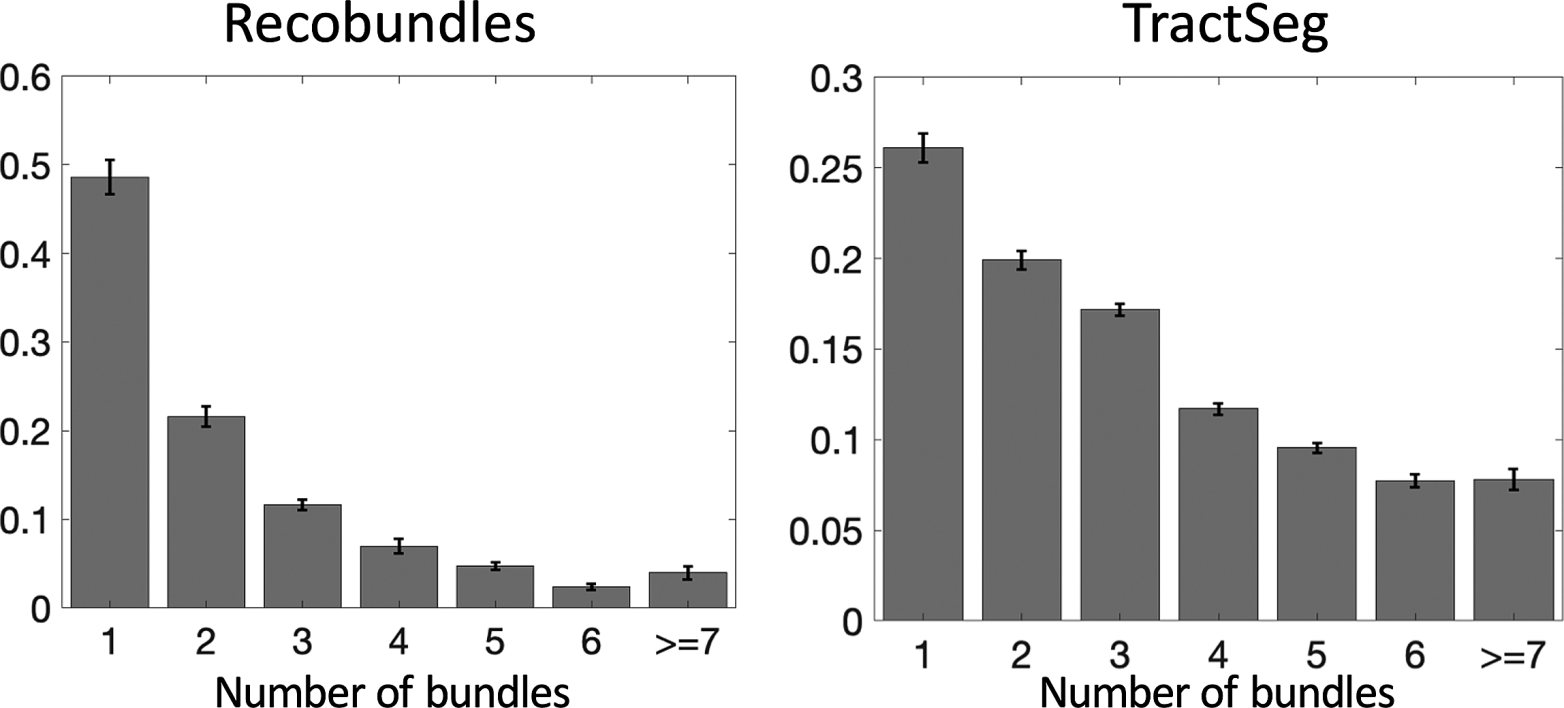
Number of bundles assigned to fixels in the brain, averaged across the population. Most fixels had greater than one bundle traversing through their designated orientation. Note that fixels which were assigned to 0 bundles are not shown. *Y*‐axis is shown as a fraction of fixels. Error bars represent variation across the studied population

Investigating fixels throughout the white matter, we first ask *what is the prevalence of multi‐bundle fixels*? Figure 4 shows population‐averaged maps of the number of bundles assigned to each fixel, visualized in coronal, sagittal, and axial views, for both TractSeg bundles (top) and Recobundles (bottom). Most noticeably, most fixels in the white matter, in all orientations, are associated with multiple bundles. In fact, many regions have groups of oriented fixels associated with 7+ unique bundles. Figure 5 quantifies the number of bundles assigned to each individual fixel, for all subjects and averaged across the population. The results confirm the qualitative observation that most fixels contain multiple bundles converging in a given orientation, with greater than 50% of fixels in Recobundles and greater than 70% of fixels in TractSeg containing greater than a single bundle population. In general, TractSeg bundles show a higher prevalence of multi‐bundle fixels (i.e., bottlenecks) than Recobundles. In summary, a majority of fixels in the brain that contain known fiber bundles act as bottleneck regions for tractography.

### 3.2 Bottleneck locations

**FIGURE 6:**
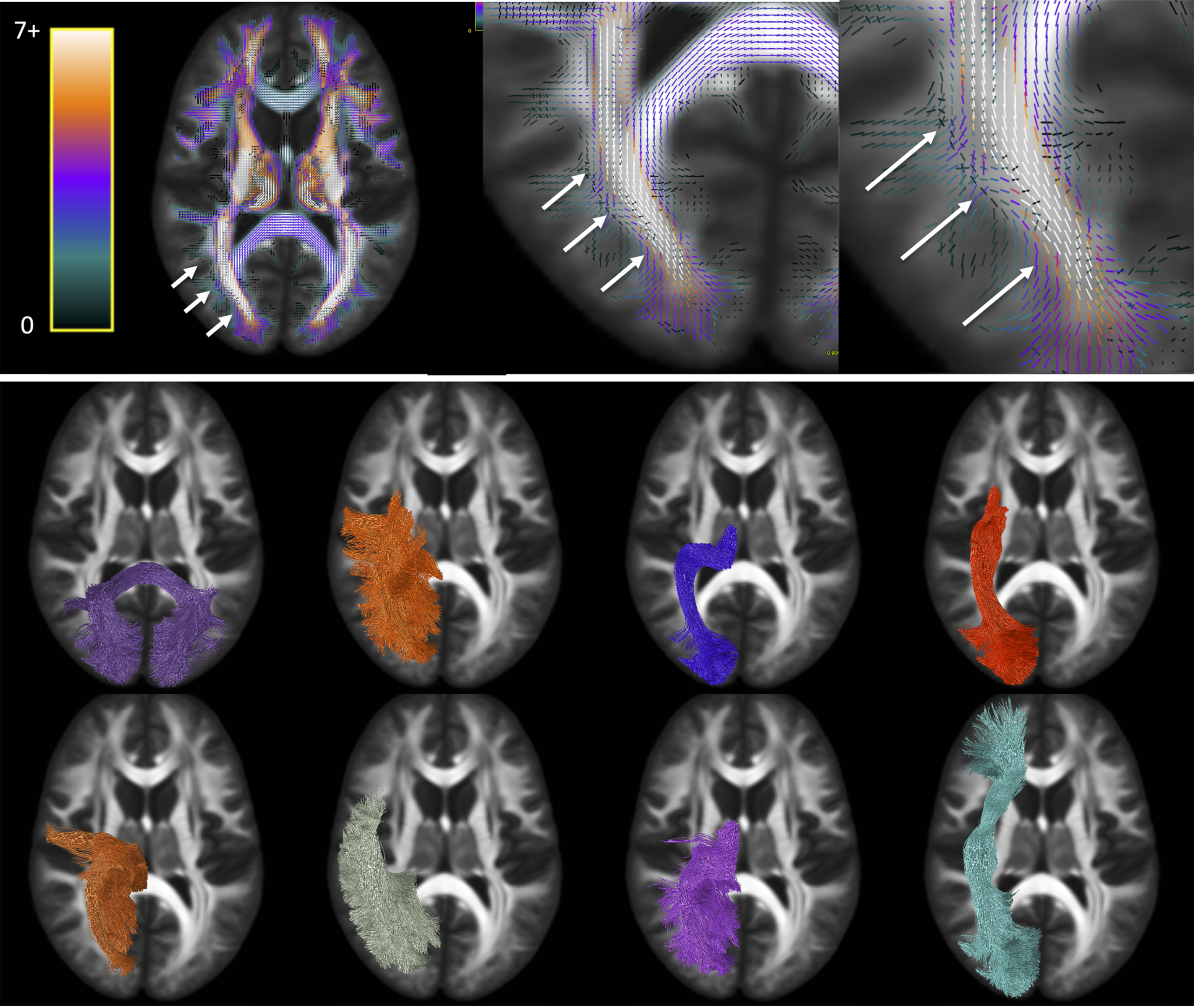
Bottleneck region in the anterior–posterior oriented white matter of the occipital lobe (arrows) contains a large number of white matter bundles with unique starting and ending connections. Colormap ranges from 0 to 7+ bundles. Pathways (derived from TractSeg) from left to right, top to bottom: CC7, ST_par, OR, ST_OCC, POPT, MdLF, T_PAR, IFO (for full names see acronyms at end of document)

**FIGURE 7:**
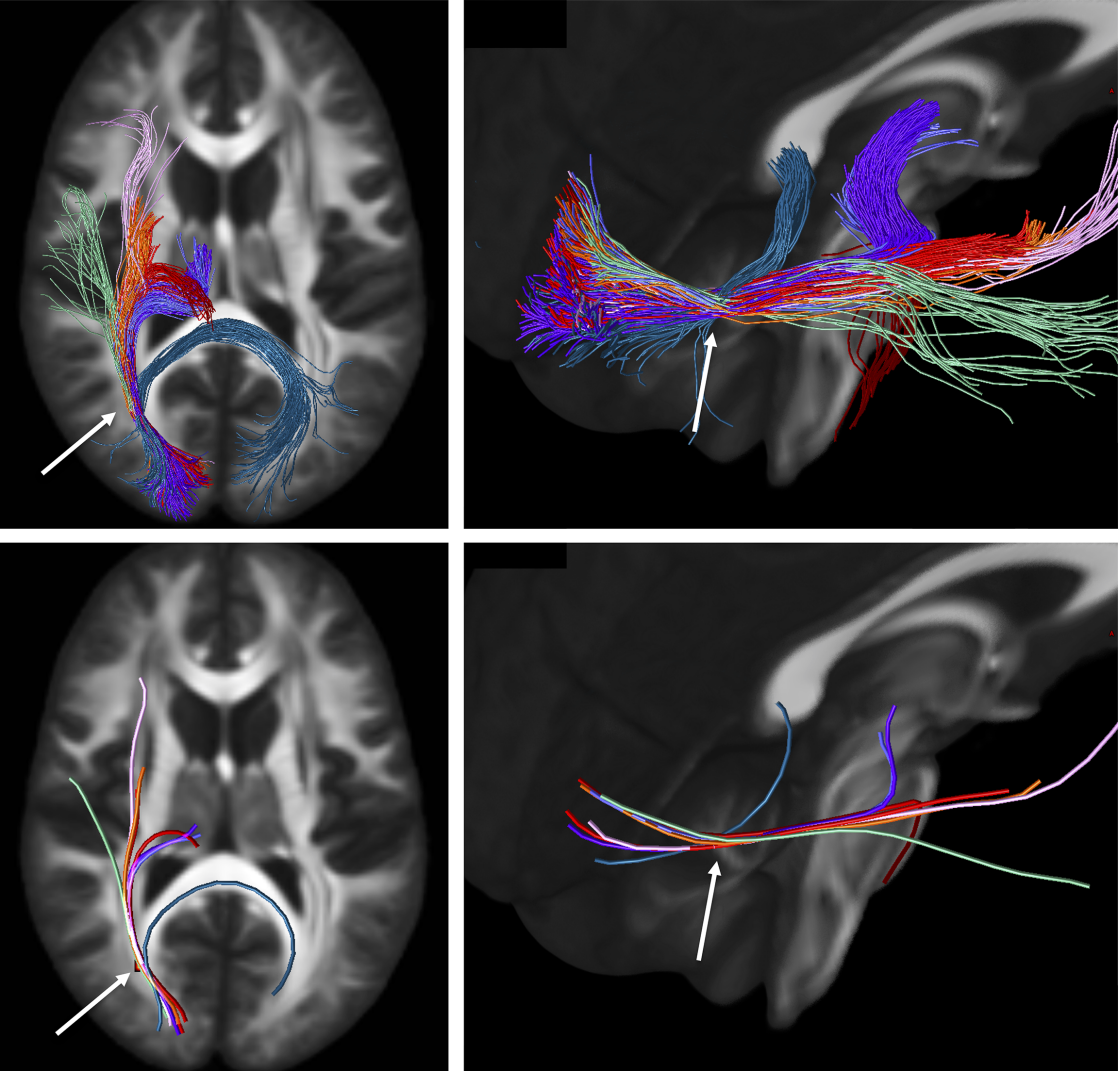
Illustration of the bottleneck in the anterior–posterior oriented white matter of the occipital lobe. Fiber bundles from Figure 5 (same color scheme) were filtered to select only streamlines (top) traversing a small 2 × 2 × 2 voxel region of interest (arrow). A single representative streamline from each sub‐bundle is also shown (bottom). This example emphasizes that streamlines belonging to many fiber bundles may traverse through the same small region, in the same orientation

**FIGURE 8:**
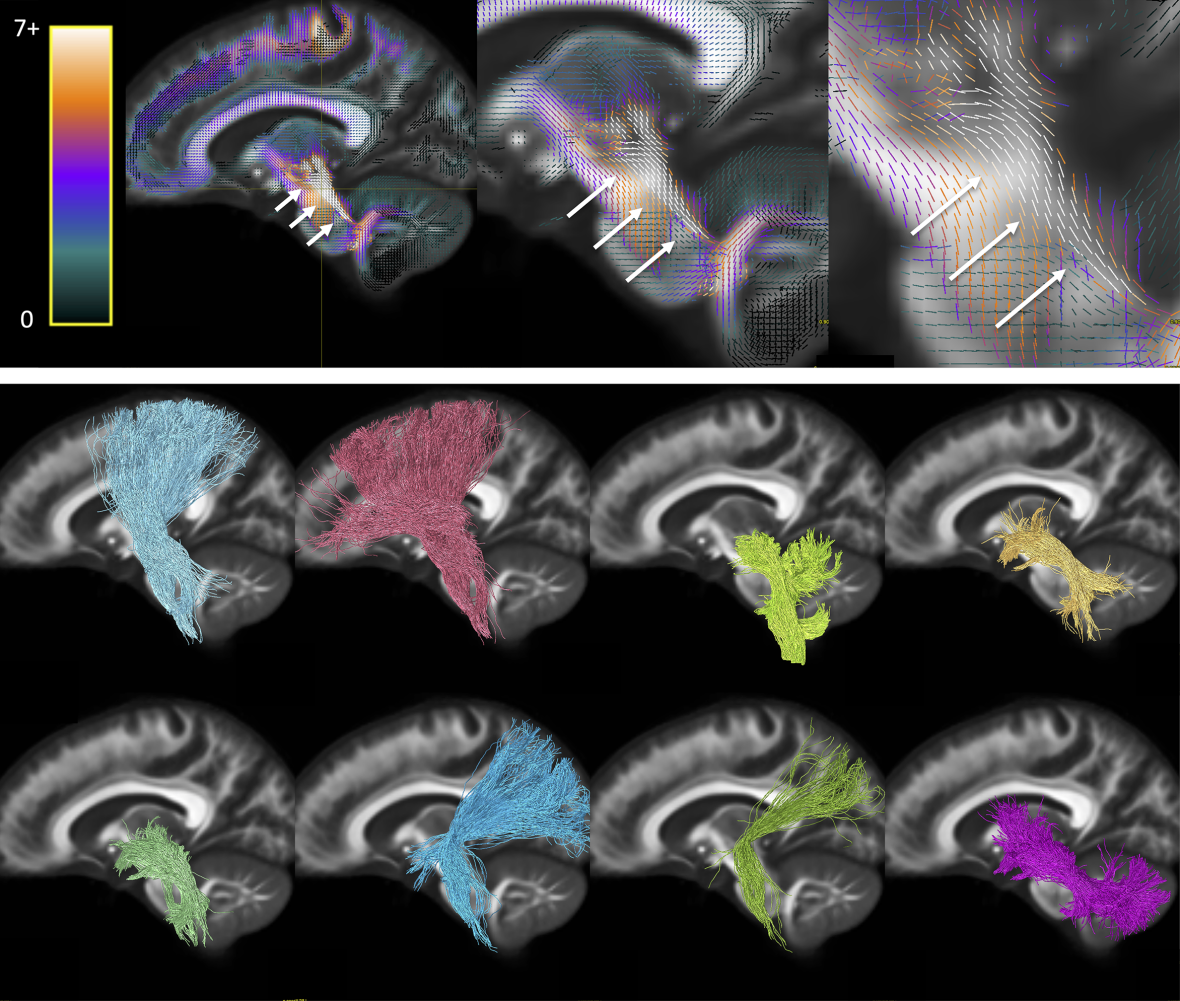
Bottleneck region in the superior–inferior oriented white matter of the brain‐stem (arrow) contains a large number of white matter bundles with unique starting and ending connections. Hot‐cold colormap ranges from 0 to 7 bundles. Pathways (derived from Recobundles) from top to bottom, left to right: CST, FPT, LL, MLL, CTT, OPT, TPT, and SCP (for full names see acronyms at end of document)

**FIGURE 9:**
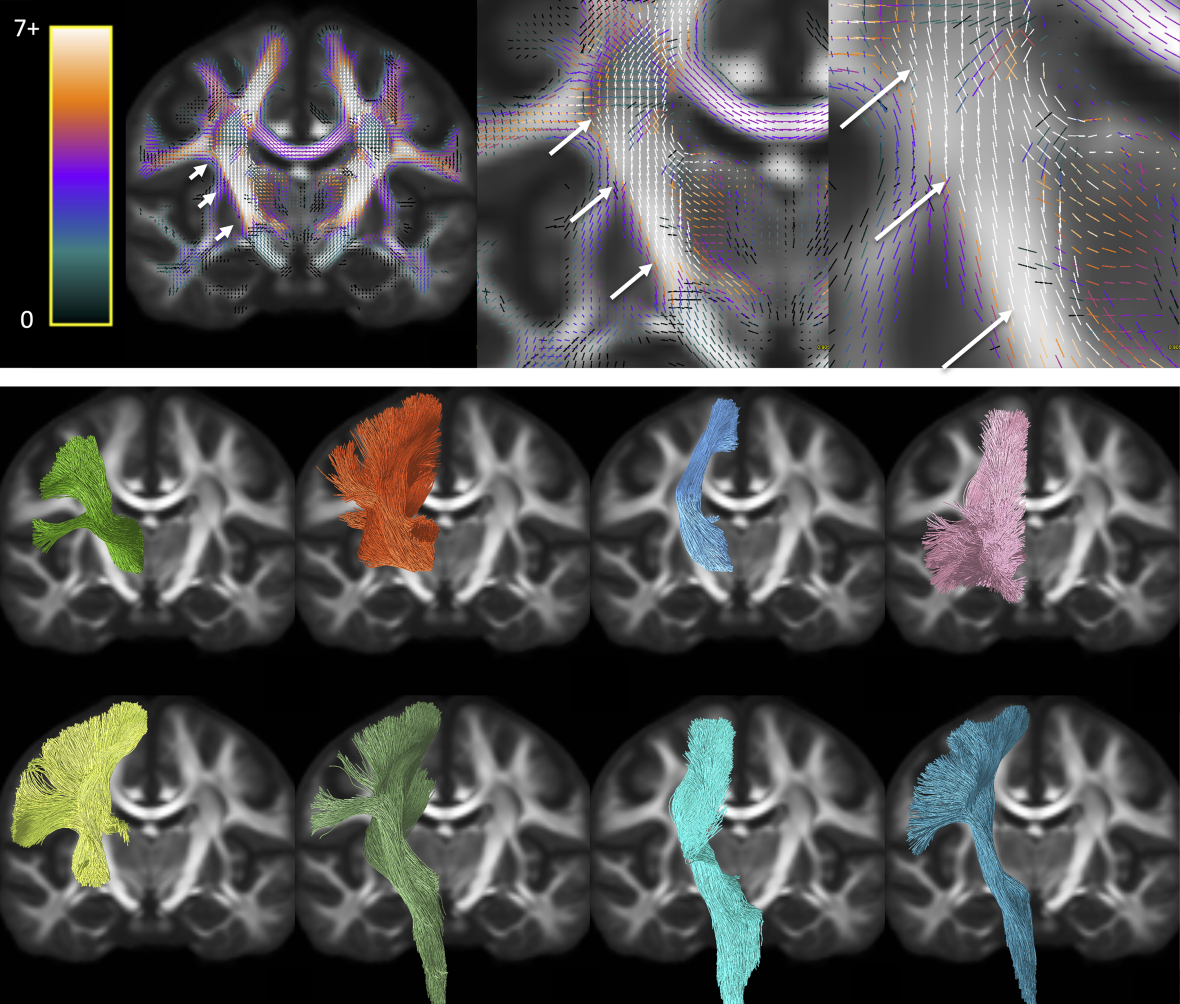
Bottleneck region in the superior–inferior oriented white matter of the internal capsule (arrow) contains a large number of white matter bundles with unique starting and ending connections. Hot‐cold colormap ranges from 0 to 7 bundles. Pathways (derived from TractSeg) from left to right, top to bottom: T_PREM, T_PAR, STR, ST_PREF, ST_POST, POPT, FPT, and CST (for full names see acronyms at end of document)

Next, we ask *where do the most important bottleneck regions occur*? These are regions in which groups of fixels with similar orientation exhibit a convergence of the largest number of pathways. Highlighted bottleneck regions, for example, 7 or more bundles that are consistent across the population, are visualized in template space in Figures 6, 7, 8, 9. In all figures, the fixels are color‐coded by the number of fibers traversing through each orientation, and exemplar bundles converging in each region are displayed.

The first displayed region with the highest number of bottlenecks (Figure 6) is located in the deep white matter of the occipital lobe oriented in the anterior–posterior direction, covering a wide dorsal‐ventral expanse and including the stratum sagittale. A large number of individual WM pathways, each with unique starting and/or ending connections, converge through this region with the same orientation, including the posterior part of the inferior fronto‐occipital fasciculus (IFOF), inferior longitudinal fasciculus (ILF), middle longitudinal fasciculus (MdLF), parieto‐thalamic and occipito‐thalamic (optic radiations, OR) connections, parieto‐striatal and parieto‐occipital pontine tract (POPT), and several subdivisions or segmentations of the striato‐cortical connections, and splenium of the corpus callosum. Thus, while most pathways terminate throughout the occipital lobe, these fibers can project to sub‐cortical nuclei, temporal or frontal lobes, or to the contralateral hemisphere as commissural fibers.

Figure 7 further highlights the bottleneck problem in this region, and illustrates how different tracts may share a similar location AND orientation, yet have unique start and end points. We filtered the previously described bundles using a single region of interest (a 2 × 2 × 2 voxel cube that is, a 2.5 mm isotropic region), and show just the streamlines from each bundle that traverse this area (top row). While the full extent of each pathway does not traverse this region, a large and coherent subset of each bundle does, all oriented in the anterior–posterior direction. To simplify the illustration, a representative streamline is shown for each filtered bundle (bottom row), exemplifying the bottleneck problem: there is a large combinatorial number of possible pathways that traverse through this voxel following this single well‐defined orientation.

The second main bottleneck region is the convergence of superior–inferior oriented fibers converging and traversing throughout the brainstem, from the mid‐brain to the medulla (Figure 8). This includes a number of ascending and descending fibers projection pathways, corticopontine fibers arising from the cortex, and cerebellar tracts. Again, fibers traversing through this region all share the same dominant orientation, yet end throughout the extent of the cortex, sub‐cortex, spinal cord, and cerebellum.

The third region with highest number of bottlenecks occurs in the superior–inferior oriented fibers of the posterior limb of the internal capsule (Figure 9). This region contains pathways such as the corticospinal tract, frontal and parietal pontine fasciculi, striato‐postcentral and striato‐precentral bundles, superior thalamic radiations toward the parietal, precentral, and postcentral cortices. While most of these fibers project from the mid‐brain and nuclei, projections cover the expanse of the parietal and frontal cortices, with many projecting onward dorsally toward the superior frontal gyrus.

### 3.3 Single‐bundle and multi‐bundle voxels

**FIGURE 10:**
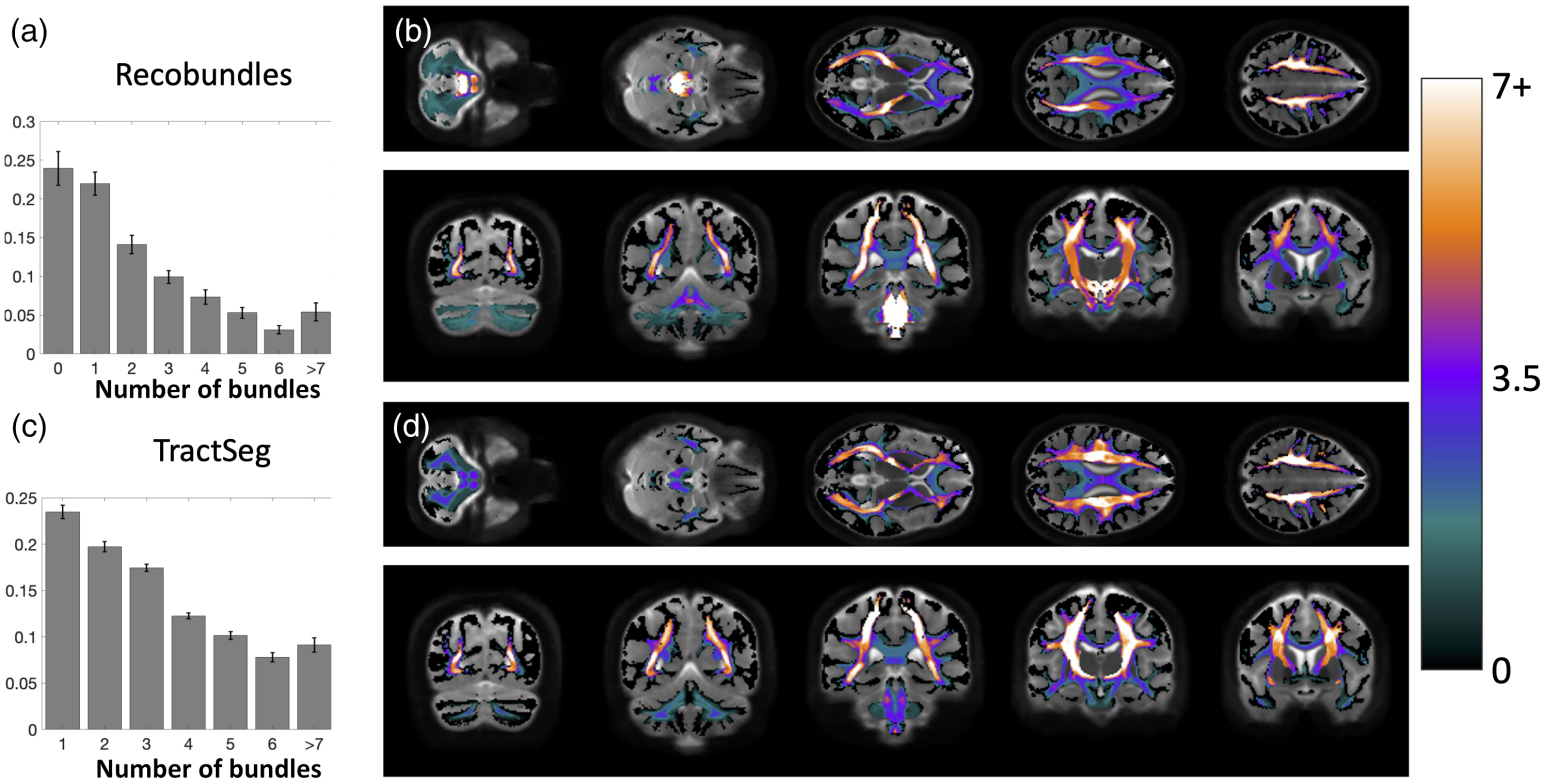
Many voxels in the white matter contain multiple known bundles. Prevalence of voxels with 1–7+ bundles averaged across the population is quantified for Recobundles (a) and visualized in template space (b), and also quantified for TractSeg bundles (c) and visualized overlaid in template space (d). Note that many voxels contain 0 bundles (i.e., are not associated with known bundles in our atlas) and are thus not quantified. Visualization in template space is mapped to a continuous color‐scale due to population‐averaging

Rather than assessing fixels, we can also ask *what is the prevalence of single‐bundle and multi‐bundle voxels*, and *where do known bundles overlap*? Thus, this overlap can contain both bundles oriented in the same direction, and different directions within a voxel. Figure 10 quantifies the number of bundles per voxel for both Recobundles and TractSeg bundles, and visualizes population‐averaged bundle overlap in the template space. By definition, a multi‐bundle fixel will reside within a multi‐bundle voxel, thus, the prevalence of multi‐bundle voxels cannot be lesser than the prevalence of multi‐bundle fixels. Consequently, it is clear that a majority of voxels in the white matter contain multiple, overlapping bundles. A number of voxels in the brain again contain as many as 7 or more unique overlapping bundles, with overlap of these bundles frequently occurring in regions that parallel the bottleneck regions— the centrum semiovale and posterior corona radiata, and also the posterior limb and retrolenticular limb of the internal capsule. While, the prevalence of multi‐bundle fixels is 51 and 74%, for Recobundles and TractSeg, respectively, the prevalence of multi‐bundle voxels is 76 and 77%—again, most white matter voxels contain overlap of multiple bundles.

### 3.4 Single‐fixel and multi‐fixel voxels

**FIGURE 11:**
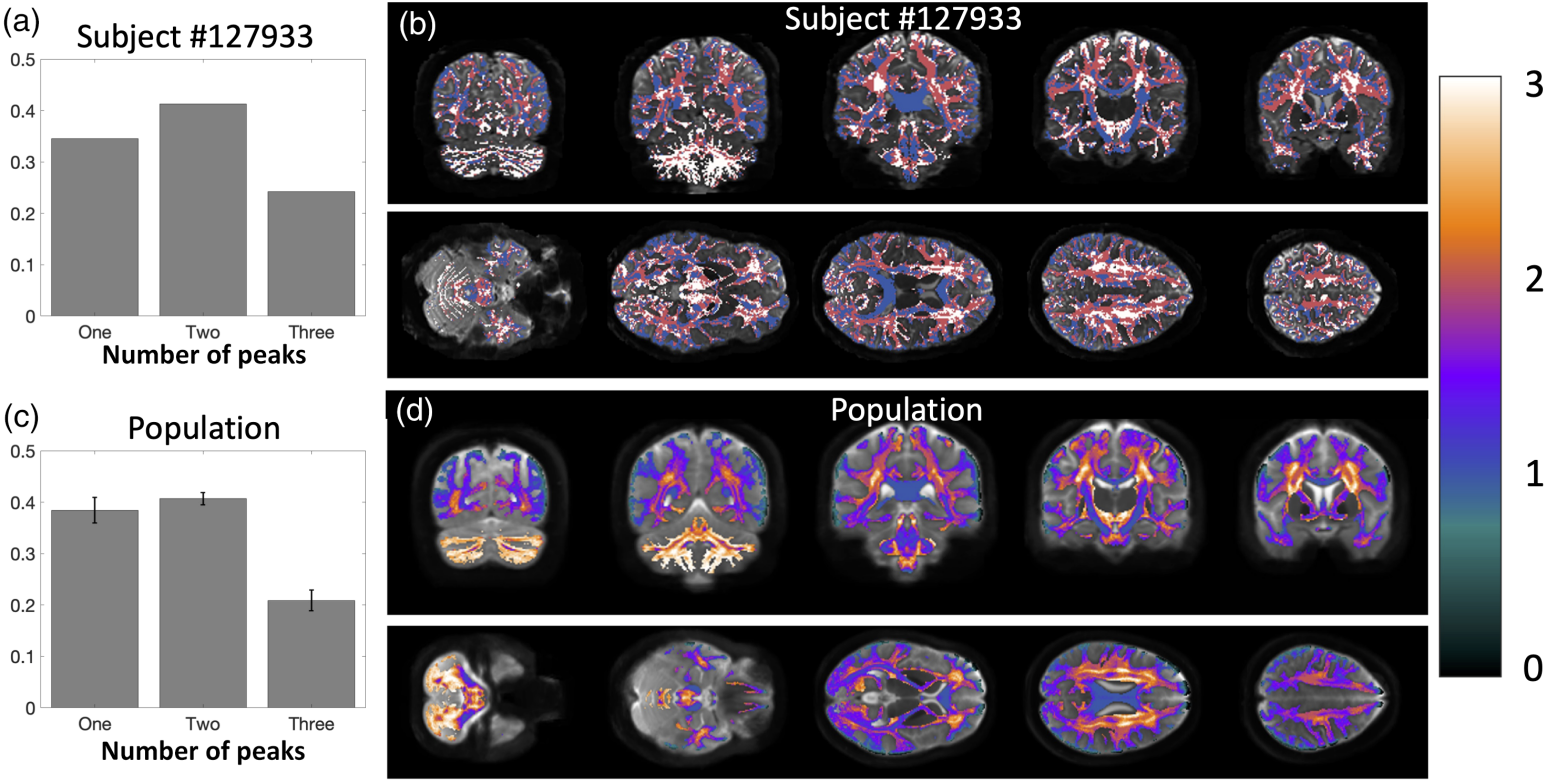
There is a high prevalence of multi‐orientation voxels throughout the brain. Prevalence of voxels with one fixel, two fixels, and three fixels is quantified for a single subject (a) and visualized overlaid on an anatomical image (b) and also averaged across the population (c) and visualized overlaid across the population template (d). Note that the number of fixels is discrete on a single subject but continuous when averaged across all subjects

Finally, we ask *what is the prevalence of single‐fixel and multi‐fixel* voxels (i.e., the prevalence of the “crossing fiber problem”), and *where do single‐ and multi‐fixel voxels occur*? Figure 11 quantifies and visualizes the prevalence of multi‐fixel voxels in both an individual subject and across the population. In agreement with previous literature, our results show that a majority of voxels in the white matter (>60%) contain multi‐fixel voxels. These voxels are prevalent throughout the entire white matter, with more complex (e.g., >2 fixels) crossings in the centrum semiovale and cerebellum. Voxels with only a single fixel are prevalent in the corpus callosum and internal capsule, as well as near the crowns of various gyri.

One particularly interesting finding is that many of the identified bottlenecks above are often associated with single‐fixel voxels—the internal capsule, mid‐brain, and less frequently in the deep white matter of the occipital lobe. For example, within voxels that have only one dominant orientation (i.e., one fixel, most have greater than one known bundle passing through that orientation, with 63 and 72% of traditionally single fiber regions containing multiple bundles.

## 4 DISCUSSION

In this study, we investigated the prevalence and locations of bottleneck regions in the brain, which present obstacles in our ability to build anatomically correct maps of the human brain using diffusion tractography.

### 4.1 Bottleneck prevalence and the problem

We find that not only do a majority of voxels contain multiple bundles, but also that a majority of individual fixels are associated with multiple bundles. As much as 50–70% of fixels in the brain contain multiple fiber bundles traversing through them. This is based on the use of “well‐known” bundles, representing only a lower‐bound that can only go up as we understand and map all existing bundles in the brain. The convergence of bundles into a nearly parallel funnel, and subsequent convergence, may lead to a combinatorial number of possible pathways that tractography algorithms may choose to take, leading to the generation of possibly anatomically nonexistent pathways. While this may not present a problem for bundle‐specific tractography with the use of manually placed priors (Chamberland et al., 2017), bundle templates (Hansen et al., 2020; Rheault et al., 2019), or machine learning (Poulin, Jörgens, Jodoin, & Descoteaux, 2019a; Wasserthal et al., 2018a; Wasserthal, Neher, & Maier‐Hein, 2018b), mapping the entirety of the human connectome ultimately strives to map *all* of the unique bundles of the brain. The identified bottleneck regions clearly present obstacles to this mapping when the true connections are not known a priori. From a whole‐brain connectome, streamlines which pass through these fixels without explicit knowledge of their existence should be suspected to be false positive connections generated by the process. Our results suggest that a majority of white matter acts as a bottleneck for tractography, hence, a large majority of streamlines derived from modern tractography algorithms are susceptible to this challenge. While structural connectomes have been used to make new discoveries about disease, and the brain in general, we caution against the direct interpretation of tractography measures as a proxy for structural connectivity or existence of structural connections. Over and above problems created in connectomics studies, this also highlights the problems of proposing the existence of a new or unique pathway from diffusion MRI alone, even if the pathway is reproducible across scans and subjects. We posit that orthogonal information in the form of blunt dissection, tracers in animals, and alternative contrasts is necessary for inferences, which demand highly specific tractography. From the neuroscientist perspective, this study suggests careful interpretation of either study of connectomes or bundle‐specific analysis. Even careful virtual dissection of specific fiber pathways will have partial volume effects throughout large extents of the bundle, hindering accurate quantitative assessment of location, size, and shape. Moreover, attributing features to this bundle (e.g., microstructural features), or along this bundle, will be possibly biased due to the existence of multiple bundles per voxel and per fixel. Similar caution is needed for clinical applications, for example in neurosurgical settings where the connections of a specific anatomy, or location of a specific bundle, require high specificity.

This bottleneck problem has further implications on quantitative tractography, or *tractometry* (Bells et al., 2011 *)*, where the along‐tract profile of measures along multiple tracts allows a comprehensive characterization of white matter. Most methods map voxel‐wise values along the tract (such as FA; Yeatman, Dougherty, Myall, Wandell, & Feldman, 2012), which we know are affected by crossing fibers, while recent studies map fixel‐wise values (such as apparent fiber density), along the tract (Chamberland et al., 2019, 2021). Yet, we show here that these measures are still not yet truly specific to one bundle. Finally, studies that use global methods of filtering or quantification (Daducci, Dal Palu, Lemkaddem, & Thiran, 2015; R. E. Smith et al., 2013) can map an average streamline‐specific estimate of diffusion or relaxometry along the tract (Barakovic et al., 2021), however, these will still be affected by partial volume at different points along the streamline profile. The ramifications of this work on diffusion tractography are also not specific to the reconstruction method used in this study, spherical deconvolution. There are several methods that may be used to arrive at fixel‐based descriptions of orientation and to quantify fiber‐specific information within a voxel, both model‐free and those based on diffusion multi‐compartment models (Dhollander et al., 2021). While these may vary in ability to resolve fiber orientation, these results are expected to generalize across methods with different *angular* resolution. We expect these to further generalize to different *spatial* resolutions, as the data used here is much higher resolution than is typical in clinical and research diffusion studies, and partial volume effects across pathways exist at resolutions well‐beyond preclinical imaging abilities (Ambrosen et al., 2020; Schilling et al., 2017).

### 4.2 Overcoming the bottleneck challenge

Here, we propose that characterizing and describing the prevalence of this problem should lead to the development of methods, which may alleviate or overcome these obstacles, much like for the crossing fiber problem. It is likely that advances in several fields will be needed to mitigate this problem. First, better and more advanced anatomical priors that will help tractography traverse bottlenecks. These priors may be elucidated through ex vivo histology, high resolution postmortem diffusion MRI, or functional contrast (functional MRI or electrophysiology) that can reveal insight into fundamental properties of white matter organization, that may include shape, curvature, connection densities, or rules of connectivity that axons follow—all of which might be replicated in the streamline propagation or filtering process. Current examples include investigations into cortical folding and anatomically‐informed curvatures (Schilling, Gao, et al., 2018; St‐Onge, Daducci, Girard, & Descoteaux, 2018), or quantifications and replication of geometric properties of the brains fiber pathways (Aranda, Rivera, & Ramirez‐Manzanares, 2014; Galinsky & Frank, 2016; C. M. W. Tax et al., 2017; Wedeen et al., 2012). Second, incorporation of microstructural or mesostructural information or diffusion properties, at the scale of voxels, fixels, or streamlines allows the incorporation of nonlocal information into the tractography process and may help traversal of bottlenecks. This may be in the form of alternative diffusion‐derived properties (Daducci et al., 2015; Girard et al., 2017; Ocampo‐Pineda et al., 2021), and implemented in a global or semi‐global approach, that attempt to modify tractography so that local streamline densities become consistent with that described by the image data. For example, recently described microstructure‐informed tractography (Girard et al., 2017) may be able to utilize microstructural features of individual bundles to overcome this problem. If a streamline enters a bottleneck region, which contains a mixture of microstructure features of many bundles, then upon exiting the bottleneck region, the appropriate direction to continue may be informed by the bundle‐specific microstructural information. However, care must be taken in this process because we show that neither the voxel, nor the fixel, carries information specific to just a single bundle of interest, and it is unknown whether a bundle is expected to have consistent microstructure properties along its entire length. Finally, machine learning techniques that can integrate microstructural information and also integrate the notion of streamline history or anatomical priors into the prediction process show promise in mitigating tractography challenges (Poulin et al., 2019a).

### 4.3 Bottleneck locations

We have described the highest bottleneck regions in this study. Specifically, we highlighted the deep white matter of the occipital lobe, the brainstem, and the internal capsule. These regions included a number of association, projection, and commissural fibers, all with unique trajectories and fundamentally different structural connections. While these were the most visually apparent “hot spots,” it is clear that a majority of fixels in the brain are associated with multiple white matter fiber pathways. From a tractography perspective, these regions may cause ambiguous connectivity estimates, yet, anatomically, these locations may have significant functional relevance, representing the intersection or merging of the many anatomo‐functional highways of the brain.

Importantly, these bottlenecks are almost certainly an underestimation of the true prevalence and extent of this problem. First, by utilizing diffusion data high spatial resolution (HCP data), we have minimized partial volume effects, particularly in the case where voxels/fixels may only share bundles at the edge of bundles. Second, the high SNR, high angular resolution, and high *b*‐value multi‐shell data allows high angular resolution, resulting the ability to more accurately discriminate multiple fiber populations per voxel, and again, minimizing the association of bundles to one fixel. Moreover, we choose segmentation techniques, which reconstruct only known anatomical pathways of the brain (72 and 66 bundles, respectively) for which there is broad agreement on their existence. Several other segmentation techniques exist which suggest the existence of a much greater number of unique pathways in the brain, however we have chosen to perform a conservative estimation, in addition to a conservative thresholding of density and/or streamlines to highlight the prevalence of this problem. Thus, these numbers are derived from a conservative estimate of the complexity of the brain, and represent the bare minimum of the number of convergent bundles. However, the absolute quantification itself may be biased toward the pathways from the chosen techniques and the atlases these are based upon. Additionally, while the bundles defined by these techniques have proven reliability, they are not themselves immune to the problems posed by bottlenecks. It is possible that improvements in tracking will refine the definitions of the bundles, as well as identify new pathways; while this may change estimates of the number of bundles in a given fixel, it won't eliminate the basic feature of the coincidence of bundles in common pathway segments.

### 4.4 Crossing fiber problem

We additionally confirm findings from previous studies which indicate that most voxels have multiple fixels (in our case, the FOD has multiple peaks). Previous studies have estimated anywhere from 30 to 90% of the white matter of the brain contains “crossing fibers” (Behrens et al., 2007; Descoteaux et al., 2009; Jeurissen et al., 2013), estimates which vary with signal to noise ratio, diffusion sensitivity, and image resolution (Jeurissen et al., 2013; Schilling et al., 2017). This suggests that most *voxels* have more than one bundle traversing its location. Over and above this, we find that most *fixels within a voxel* have more than one bundle traversing in its direction. Thus, even solving the crossing fiber problem does not solve the bottleneck problem that may cause a large number of ambiguous, false positive pathways.

### 4.5 Adding to known atlases

While the primary aim of this study was to characterize bottlenecks, in which >1 bundle passes through a fixel, we made the interesting ancillary observation (Figure S1) that many fixels within the white matter were not associated with any bundles defined in our utilized atlases. We note that these “zero‐bundle” fixels were not included in the analysis because there is a lack of information regarding these orientations. While many of these could be spurious fixels or isolated voxels in white matter, larger expanses of coherently oriented fixels could be regions, which may represent underexplored white matter pathways which can eventually be added to our repertoire and collection of bundles. While some regions are easily explained, for example due to a lack of TractSeg bundles in the cerebellum, other regions occur both along and across several gyral blades, as well as the relatively underexplored system of U‐fibers and local association fibers. Further reasons for this could be the thresholding of densities and streamlines and parameter configurations for the bundle segmentation techniques. Additional atlases or bundle segmentation procedures may include pathways through many of these regions, which would solve the missing‐bundle problem, but would likely also increase the prevalence of bottlenecks.

This is a potential limitation of the current study—the choice of atlases (bundle segmentation procedures). We have purposefully chosen to only include pathways for which there is broad agreement on their existence, and techniques, which have been validated and well‐utilized in the field. There would also be the potential to define pathways and bottleneck regions using whole‐brain connectivity, or atlases which are derived from clusters or reproducibility of large datasets (Guevara et al., 2012; Ros, Gullmar, Stenzel, Mentzel, & Reichenbach, 2013; Siless, Chang, Fischl, & Yendiki, 2018), however, these are potentially confounded by the bottleneck problem itself (i.e., contain false positive, yet reproducible, pathways), and an extensive comparison of algorithms and segmentation methods is beyond the scope of the current study.

### 4.6 “Single fiber populations” and microstructure

In addition to tractography, the diffusion signal that results from a single fiber population (i.e., the fiber response function) has applications toward tissue microstructural modeling. The response function can be used to estimate the FOD and is inherently sensitive to tissue microstructural properties including diffusivities, compartment sizes, and orientation dispersions. Typically, this response function is derived from studying regions of low complexity, specifically, regions that are considered single fiber populations and contain only a single peak in the FOD (Dhollander et al., 2016; C. M. Tax, Jeurissen, Vos, Viergever, & Leemans, 2014; Tournier et al., 2007). Here, however, we can see that even if a voxel or region contains a dominant orientation and high anisotropy, a majority of these regions are composed of multiple, distinct fiber pathways, that may have varying densities, sizes, and distributions of axons. For example, if we were to use the traditional definition of a “single fiber population” (i.e., our single‐fixel voxel) we would find that only 35 and 27% contain just a single fiber bundle passing through them (Figure S2). Thus, these so‐called single fiber regions are very often multi‐bundle regions. Truly single‐fiber and single‐bundle regions are rare, even with our limited selection of known bundles used in this study, and in our case, occur in the cingulum and specific gyral blades (Figure S3). While some works show that biological differences between fiber populations are negligible in the response function formulism (Christiaens et al., 2020), there is some evidence that the response functions do vary across pathways (Howard et al., 2019; Schilling, Gao, et al., 2019; C. M. W. Tax et al., 2015), which may lead to variation in estimates of FODs and subsequent microstructure.

### 4.7 Nomenclature

Here, we have also introduced slightly different nomenclature than past literature. As described above a single‐fixel voxel has traditionally been called a single‐fiber voxel, whereas a multi‐fixel voxel has been called crossing‐fiber voxel. Clearly, a voxel with only one orientation is not limited to only containing the presence of fibers from a single bundle, hence the new description based on fixels rather than fibers. Additionally, a fixel has sometimes been interpreted as a “specific fiber bundle within a specific voxel” (Genc et al., 2018; Honnedevasthana Arun, Connelly, Smith, & Calamante, 2021; D. A. Raffelt et al., 2015, 2017), yet we have shown again that a fixel, is not limited to a single specific fiber bundle (based on our definition of a fiber bundle), and in fact, likely contains axons from several bundles. However, more generally, a “fixel” is a contraction of a “fiber element” and can inherently contain and support a nonzero attribution to multiple bundles (Dhollander et al., 2021), where the term fiber simply refers to all fibers pointing in a particular direction. This study does not negate, nor minimize, the use and definition of a fixel, but emphasizes the importance of considering partial volume effects within fixels, and clarity when discussing our use of the word “bundle” versus a “fiber bundle element” which describes the contents of a voxel pointing in a specific direction.

We are not proposing to change the use and discussion of these elements throughout the field, but rather chose clarifying nomenclature to remove ambiguity in this specific study. While the definition, and use of the word, *bundle* is fairly noncontroversial, it is important to point out that different sets of bundles used can change the prevalence of this problem. We use the term to represent sets of streamlines that start and end in locations generally belonging to the same brain structural or functional territories, and use only those that represent macroscopic pathways known to exist in the human brain. One can perform sub‐divisions of many bundles (e.g., lateral/middle/medial thirds of CST), or even consider all streamlines which cluster together in some predetermined way (Garyfallidis et al., 2018; Guevara et al., 2012; Ros et al., 2013; Siless et al., 2018; F. Zhang et al., 2018) as bundles—which would in fact meet our definition of a bundle—but would lead to an over‐estimation of the problem. While we did not perform blind clustering, in some cases we did utilize divisions of some pathways (e.g., TractSeg defines SLF I, II, and III) which is justified because these have unique, distinct, connections and different functional roles, and must be able to be unambiguously resolved for anatomically accurate studies of structure and function.

## 5 CONCLUSION

In this work, we investigated the prevalence of bottleneck regions, or where multiple white matter pathways of the brain converge and subsequently diverge. Our results indicate that most white matter contains multiple overlapping bundles, and individual orientations within a voxel are associated with multiple bundles. These findings have profound implications for tractography analysis, which aims to map unknown connections across the brain, and strengthen the awareness of limitations or challenges facing these image processing techniques.

## Supporting information


**Figure S1** Template space fixels in which zero fiber bundles are observed for Recobundles (a) and TractSeg (b).
**Figure S2**. Within voxels that only have one dominant orientation (i.e., one fixel), most have greater than one known bundle passing through that voxel. Bar plots show the number of bundles assigned to single fixel voxels for Recobundles and TractSeg algorithms.
**Figure S3**. Template space fixels in which a single fiber bundle is observed for Recobundles (a) and TractSeg (b).

## Data Availability

Data were provided [in part] by the Human Connectome Project, WU‐Minn Consortium (Principal Investigators: David Van Essen and Kamil Ugurbil; 1U54MH091657) funded by the 16 NIH Institutes and Centers that support the NIH Blueprint for Neuroscience Research; and by the McDonnell Center for Systems Neuroscience at Washington University. All resulting voxel‐wise and fixel‐wise bundle overlaps, for both TractSeg and Recobundles, is available upon request.

## Appendix

The bundles resulting from each bundle‐segmentation pipeline are given as a list below.

Recobundles:

Anterior Commissure (AC); Arcuate Fasciculus L/R (AF); Acoustic Radiation L/R (AR); Frontal Aslant Tract L/R (AST); Cerebellum L/R (CB); Corpus Callosum Mid (CC_Mid); Corpus Callosum Major (CC_ForcepsMajor); Corpus Callosum Minor (CC_ForcepsMinor); Corticospinal tract L/R (CST); Corticostriatal pathway L/R (CS); Central Tegmental Tract L/R (CTT); Corticothalamic pathway L/R (CT); Cingulum L/R (C); Dorsal Longitudinal Fasciculus L/R (DLF); Extreme Capsule L/R (EMC); Frontopontine Tract L/R (FPT); Fornix (FX); Inferior Cerebellar Peduncle L/R (ICP); Inferior Fronto‐Occipital Fasciculus L/R (IFOF); Inferior Longitudinal Fasciculus L/R (ILF); Lateral Lemniscus L/R (LL); Middle Cerebellar Peduncle (MCP); Medial Longitudinal Fasciculus L/R (MLF); Medial Lemniscus L/R (ML); Middle Longitudinal Fasciculus L/R (MdLF); Occipito Pontine Tract L/R (OPT); Optic Radiation L/R (OR); Posterior Commissure (PC); Parieto Pontine Tract L/R (PPT); Rubrospinal Tract L/R (RST); Superior Cerebellar Peduncle (SCP); Superior Longitudinal Fasciculus L/R (SLF); Spinothalamic Tract L/R (STT); Temporopontine Tract L/R (TPT); Uncinate Fasciculus L/R (UF); Vertical Occipital Fasciculus L/R (VOF).

TractSeg:

Arcuate fascicle L/R (AF); Anterior Thalamic Radiation L/R (ATR); Commissure Anterior (CA); Rostrum (CC_1); Genu (CC_2); Rostral body – Premotor (CC_3); Anterior midbody ‐ Primary Motor (CC_4) ; Posterior midbody ‐ Primary Somatosensory (CC_5); Isthmus (CC_6); Splenium (CC_7); Cingulum L/R (CG); Corticospinal Tract L/R (CST); Middle longitudinal fascicle L/R (MLF); Fronto‐pontine tract L/R (FPT); Fornix L/R (FX); Inferior cerebellar peduncle L/R (ICP); Inferior occipito‐frontal fascicle L/R (IFO); Inferior longitudinal fascicle L/R (ILF); Middle cerebellar peduncle (MCP); Optic radiation L/R (OR); Parieto‐occipital pontine L/R (POPT); Superior cerebellar peduncle L/R (SCP) ; Superior longitudinal fascicle I L/R (SLF_I); Superior longitudinal fascicle II L/R (SLF_II); Superior longitudinal fascicle III L/R (SLF_III); Superior Thalamic Radiation L/R (STR); Uncinate fascicle L/R (UF); Thalamo‐prefrontal L/R (T_PREF); Thalamo‐premotor L/R (T_PREM); Thalamo‐precentral L/R (T_PREC); Thalamo‐postcentral L/R (T_POSTC); Thalamo‐parietal L/R (T_PAR); Thalamo‐occipital L/R (T_OCC); Striato‐fronto‐orbital L/R (ST_FO); Striato‐prefrontal L/R (ST_PREF); Striato‐premotor L/R (ST_PREM); Striato‐precentral L/R (ST_PREC); Striato‐postcentral L/R (ST_POSTC); Striato‐parietal L/R (ST_PAR); Striato‐occipital L/R (ST_OCC).
