# Supplementary material for: Prevalence of white matter pathways coming into a single white matter voxel orientation: The bottleneck issue in tractography
Source: Hum Brain Mapp. 2021 Dec 17;43(4):1196–213. doi: 10.1002/hbm.25697 (PMC8837578; doi:10.1002/hbm.25697)
Supplement: Supplementary file 1 — Figure S1 Template space fixels in which zero fiber bundles are observed for Recobundles (a) and TractSeg (b). Figure S2. Within voxels that only have one dominant orientation (i.e., one fixel), most have greater than one known bundle passing through that voxel. Bar plots show the number of bundles assigned to single fixel voxels for Recobundles and TractSeg algorithms. Figure S3. Template space fixels in which a single fiber bundle is observed for Recobundles (a) and TractSeg (b). [file HBM-43-1196-s001.docx]

Supplementary Figure s

*
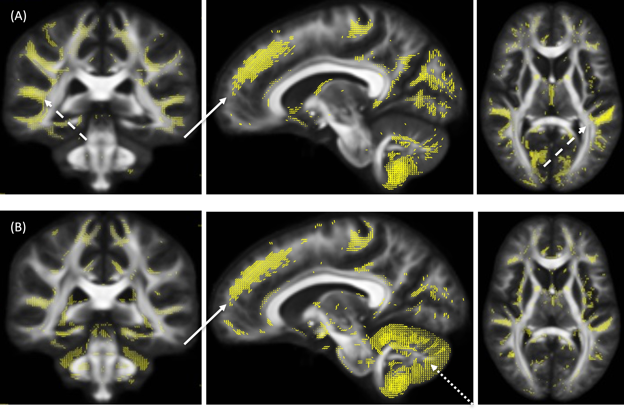
*

Supplementary Figure 1. Template space fixels in which zero fiber bundles are observed for Recobundles (A) and TractSeg (B).

*
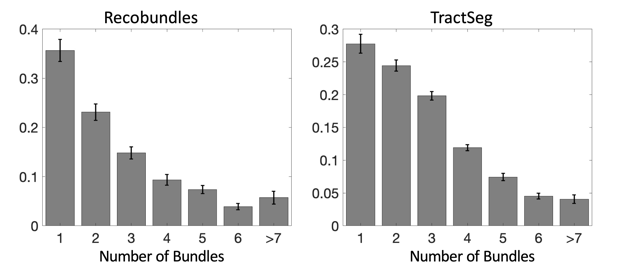
*

Supplementary Figure 2. Within voxels that only have one dominant orientation (i.e., one fixel), most have greater than one known bundle passing through that voxel. Bar plots show the number of bundles assigned to single fixel voxels for Recobundles and TractSeg algorithms.

*
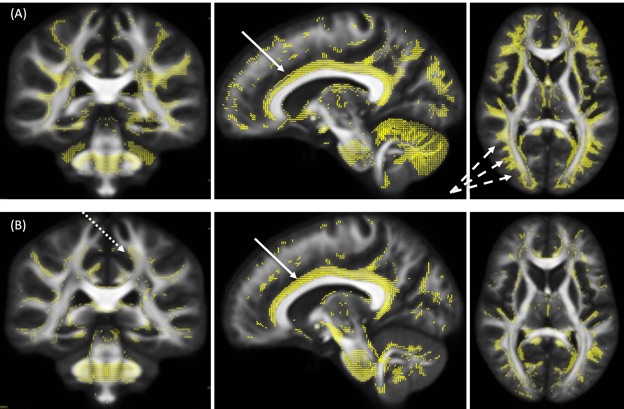
*

Supplementary Figure 3. Template space fixels in which a single fiber bundle is observed for Recobundles (A) and TractSeg (B).
